# Profiling of CD63 and EpCAM Membrane Proteins of Extracellular Vesicles on Tannic Acid-Coated Magnetic Beads Using Conventional Flow Cytometry

**DOI:** 10.3390/ijms262311324

**Published:** 2025-11-23

**Authors:** Ekaterina Moiseeva, Igor Sergeev, Vasiliy Chernyshev, Olga Zaborova, Daria Kohzevnikova, Alexander Yakovlev, Olesya Kuznetsova, Alexey Tryakin, Aleksei Komlev, Dmitry Gorin, Alexey Yashchenok

**Affiliations:** 1 Skolkovo Institute of Science and Technology, Skolkovo Innovation Center, 121205 Moscow, Russia; ekaterina.moiseeva@skoltech.ru (E.M.); igor.sergeev@skoltech.ru (I.S.); v_chernyshev@oparina4.ru (V.C.); d.kozhevnikova@skoltech.ru (D.K.); d.gorin@skoltech.ru (D.G.); 2 National Medical Research Center for Obstetrics, Gynecology and Perinatology Named after Academician V.I. Kulakov, 117997 Moscow, Russia; 3 Faculty of Chemistry, Moscow State University, 119991 Moscow, Russia; olya_z_88@mail.ru; 4 Moscow Research and Clinical Center for Neuropsychiatry, 115419 Moscow, Russia; al_yakovlev@ihna.ru; 5 Department of Functional Biochemistry of Nervous System, Institute of Higher Nervous Activity and Neurophysiology, Russian Academy of Sciences, 117865 Moscow, Russia; 6 N.N. Blokhin National Medical Research Center of Oncology, 115522 Moscow, Russia; lessya.kuznetsova@gmail.com (O.K.); atryakin@gmail.com (A.T.); 7 Faculty of Physics, Moscow State University, 119991 Moscow, Russia; alkomlev98@yandex.ru

**Keywords:** exosomes, small extracellular vesicles, superparamagnetic beads, cancer diagnostics, liquid biopsy

## Abstract

Extracellular vesicles (EVs) are considered to be a promising tool in disease diagnosis. However, the clinical translation of EV-based liquid biopsy faces significant challenges due to the lack of inexpensive, rapid, and high-throughput methods of EV analysis. Bead-based platforms, combined with conventional flow cytometry, allow for the simultaneous capture and immunolabeling of EVs. In this study, we present a new approach based on the label-free isolation of EVs by tannic acid-coated superparamagnetic beads (TASPMB) combined with immunofluorescence detection of EV membrane proteins using flow cytometry. First, we tested the molecular profiling capabilities of the approach using EVs derived from human breast and colorectal cancer cell lines and from plasma of colorectal cancer patients to recognize the tetraspanin protein CD63 and the epithelial cell adhesion molecule (EpCAM). Subsequently, the developed approach was validated to identify proteins on EVs enriched with TASPMB from the conditioned media of SKBR3 and HT29 cell cultures without preliminary purification by a size-exclusion chromatography (SEC) column. The developed approach demonstrates a high capacity for isolating EVs and subsequently profiling of their membrane proteins, with a total assay time of approximately 2 h. The approach presented here can be a promising tool for rapid detection of EV membrane proteins using conventional instruments, such as flow cytometry.

## 1. Introduction

Extracellular vesicles (EVs) are membrane vesicles secreted by all cell types and circulating in numerous biological fluids of the human body. EVs act as messengers between cells, transporting nucleic acids and proteins [1]. Therefore, EVs can be involved in various physiological and pathophysiological processes, such as immune responses [2], tumor progression [3], viral infections [4], and others [5]. Given that all EVs are composed of proteins and lipids reflecting the biochemical composition and the status of the parent cell, as well as the high amount of EVs present in biological fluids, they are considered promising biomarkers of various diseases, including cancer [6]. In this regard, the isolation of tumor-derived EVs followed by the analysis of either EVs’ membrane proteins or EVs’ cargo would make a significant contribution both for understanding their functions and finding the way to use them in the development of minimally invasive diagnostic tools [7,8].

Methods for studying EVs are constantly evolving; however, obtaining purified and biologically active EVs from biological samples for downstream analysis remains a significant challenge. Ultracentrifugation is the most frequently used method for enriching EVs from various biological sources, such as cell culture medium, urine, and blood plasma [9]. In addition, ultrafiltration and sedimentation by polymers are also used to isolate EVs from various biological samples [10,11]. These methods, nevertheless, do not provide sufficient amounts and purity of EVs, require trained personnel, and long operation time [12]. The disadvantages can be somewhat overcome by using the size-exclusion chromatography (SEC) method. The SEC method is convenient, inexpensive, and avoids significant protein contamination or EV damage [13]. At the same time, for the molecular content discovery of EVs or direct quantification of EVs, all the above methods should be followed by additional analysis using Western blotting (WB), polymerase chain reaction, or mass spectrometry, which in turn require highly qualified specialists, long-term operation, and costly equipment.

Flow cytometry (FC) is an established method that is widely used for routine quantitative and qualitative analysis of cells; therefore, it is also considered a promising method for EVs’ study [14]. However, the application of FC for EVs’ analysis is limited because EVs with a diameter below 300 nm are not efficiently discriminated from the background signal in scattering modes [15]. The sensitivity of FC in EVs analysis can be improved by an optimized configuration of FC equipment [16] or through double labeling of EVs with protein- and lipid-specific dyes [13,17]. However, in the meantime, the above protocols require manual hardware adjustment and calibration of the FC before use, as well as a time-consuming labeling step and further EV purification from the unbound dye.

On the other hand, isolation of EVs using magnetic beads combined with conventional flow cytometry (FC) analysis is an attractive technique for the development of innovative liquid biopsy based on EV biomarker detection [18,19]. First, EVs secreted by tumor tissues can be captured from the biological samples using magnetic beads conjugated with appropriate ligands which are capable of either specifically or non-specifically interacting with EVs’ surface receptors [20]. Second, the trapped EVs can be purified by multiple washes, thus ensuring a fraction of EVs free from non-vesicular particles and contaminant proteins. A bead-based platform is highly efficient in terms of purity, providing a higher concentration of exosome-specific proteins compared to common isolation strategies [21]. Finally, analysis of EV membrane proteins can be performed immediately on the bead surface using flow cytometry (FC) and commercially available fluorescently labeled antibodies, aptamers, and peptides [22]. In this regard, platforms based on magnetic beads are beneficial both for isolation, purification, and molecular analysis of EVs, offering vast opportunities for the detection of a wide range of tumor biomarkers.

Generally, affinity capture is the most common method for isolating EVs using magnetic beads [22]. Beads conjugated with vector molecules such as antibodies, peptides, or aptamers targeted to EVs membrane markers, such as tetraspanins CD9, CD63, CD81, epithelial cell adhesion molecule (EpCAM), or the phosphatidylserine receptor (Tim4) [23] are applied for isolation of the total population of EVs. However, the isolation of distinct EV populations is often limited due to the high cost or poor reproducibility of the kits. Alternatively, EVs can be captured based on electrostatic attraction [24], dipole–dipole interaction [25,26], or hydrogen bonding [27]. In our previous work, we demonstrated that tannic acid-coated magnetic beads had a high efficiency in capturing EVs (60%) from solutions with low protein content, primarily cell culture medium [28]. TA is a natural organic polyphenolic compound containing ten molecules of gallic acid. The robust EVs’ interaction with TA is attributed to the formation of a cation-p complex between the galloyl ring and the quaternary amine group of phospholipid membranes, in addition to hydrogen bonding and hydrophobic interaction. The size and morphology of EVs in the supernatant and eluate did not change following beads’ adsorption. Furthermore, non-bound proteins can be removed from the beads’ surfaces, while EVs remain adsorbed. Considering these characteristics, tannic acid-coated beads offer a promising method for purifying and analyzing EVs.

In this study, we applied tannic acid-functionalized superparamagnetic beads (TASPMB) for enrichment of EVs from conditioned media of two humans’ colorectal cancer HT29 and breast cancer SKBR3 cell cultures. Subsequently, the captured EVs by TASPMB were analyzed using conventional flow cytometry through labeling of CD63 and EpCAM proteins in the plasma membrane of EVs. The approach demonstrated in our study can be useful for basic research that requires the rapid and reliable isolation and molecular profiling of EVs.

## 2. Results

### 2.1. Fabrication of Superparamagnetic Beads

**Figure 1:**
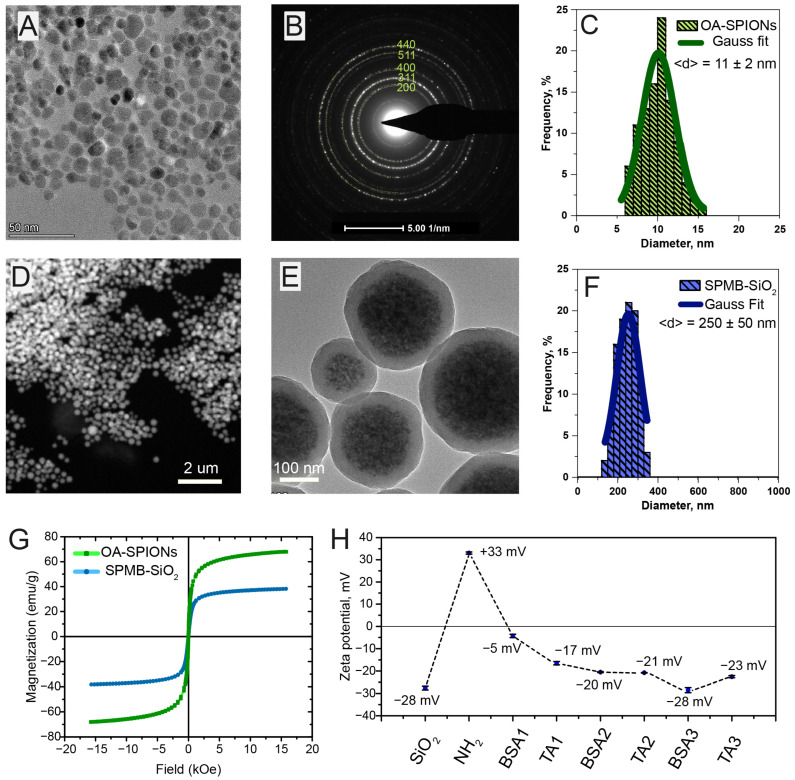
Fabrication of tannic acid (TA)-functionalized superparamagnetic beads (SPMB). (**A**) High resolution transmission electron microscopy (HRTEM) image of oleic acid-stabilized superparamagnetic iron oxide nanoparticles (OA-SPIONs). (**B**) Small-angle electron diffraction (SAED) of OA-SPIONs. (**C**) OA-SPIONs size distribution histogram. (**D**) High-angle annular dark-field high-resolution scanning transmission electron microscopy (HAADF-STEM) imaging of SPMB. (**E**) HRTEM image of SPMB. (**F**) SPMB size distribution histogram. (**G**) Magnetization loops of SPMB and OA-SPIONs at 300 K. (**H**) Zeta potential measurements of SPMB, amino-functionalized SPMB and sequential deposition of bovine serum albumin (BSA) and TA on amino-functionalized SPMB.

Superparamagnetic beads (SPMB) were fabricated by self-assembly of hydrophobic iron oxide nanoparticles encapsulated within a thin silica shell. Initially, superparamagnetic iron oxide nanoparticles (SPIONs) were synthesized by co-precipitation of divalent and trivalent iron salts in an aqueous medium. To obtain hydrophobic SPIONs for dispersion in a volatile organic solvent such as chloroform, oleic acid (OA) was used as a stabilizing agent. High resolution transmission electron microscopy (HRTEM) studies (Figure 1A) revealed nearly spherical morphology of SPIONs with relatively narrow size distribution. The selected-area small-angle electron diffraction (SAED) patterns displayed characteristic ring structures of SPIONs, which correspond to a polycrystalline structure. The presence of (200), (311), (400), (511), and (440) crystallographic planes indicates a cubic superlattice, which is common for maghemite–magnetite spinel systems (Figure 1B). This is most probably due to the presence of a non-stoichiometric mixture of maghemite and magnetite iron oxides [29]. The mean diameter of SPIONs was estimated to be 11 ± 2 nm (Figure 1C). Next, the obtained SPIONs were then organized into clusters by a self-assembly approach [30]. The hydrophobic van der Waals interactions between the alkane chains of OA and surfactant drive this process. The final step involved introducing PVP-stabilized clusters [31] into a Stober reaction to form a silica layer around the clusters. The obtained SPMBs were inspected on HRTEM using STEM and TEM modes (Figure 1D,E). The average diameter of SPMB was found to be 250 nm with a relatively narrow distribution of 50 nm (Figure 1F). The magnetic properties of SPMB were examined by the Vibrating Sample Magnetometry (VSM) method (LakeShore 7407). The saturation magnetization reached 40 emu/g (Figure 1G). Moreover, zero remnant magnetization and the absence of coercivity indicate the superparamagnetic nature of SPMB. The latter is due to the superparamagnetic origin of the hydrophobic SPIONs precursor, which has a high saturation magnetization of 61 emu/g.

### 2.2. Functionalization of Superparamagnetic Beads with Tannic Acid

The successful formation of amino groups on the surface of SPMB was confirmed by a change in zeta potential from −28 mV for SPMB to +33 mV for amino-functionalized SPMB (Figure 1H). To deposit TA on amino SPMB, we used the layer-by-layer (LbL) method. Bovine serum albumin was first deposited on amino SPMB, followed by adsorption of TA. The adsorption of BSA and TA was monitored by measuring the zeta potential of SPMB after each deposition step (Figure 1H). One can see the adsorption of the first BSA layer changes the zeta potential of amino SPMB from +33 mV to −5 mV and then to −17 mV after the deposition of TA. The zeta potential upon the adsorption of subsequent layers of BSA and TA is characterized by an overall negative value and a change in absolute value. These data confirmed successful functionalization of SPMB and are consistent with our previous results [28].

### 2.3. Isolation and Characterization of EVs

**Figure 2:**
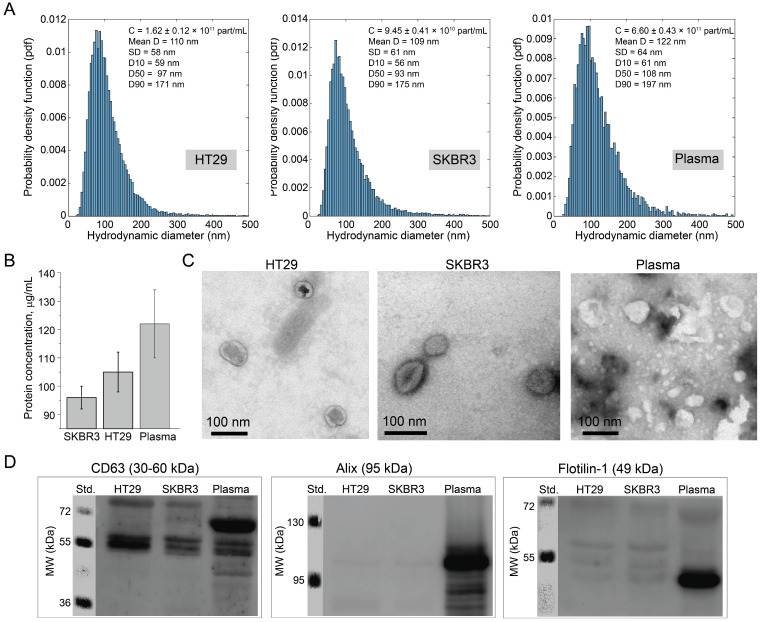
Characterization of EVs isolated from human colorectal cancer HT29 and breast cancer SKBR3 cell lines, and blood plasma of a colorectal cancer patient by a size-exclusion chromatography (SEC) column (**A**). The hydrodynamic size distribution of isolated EVs determined by the nanoparticle tracking analysis (NTA) method is presented in the form of a probability density function (PDF). Numbers in each panel represent concentration, mean diameter, and standard deviation (SD). (**B**) Protein concentration in EV samples was determined by the BCA method. (**C**) Transmission electron microscopy (TEM) images of EVs. (**D**) Western blotting of EVs indicated the relative expression of characteristic EV proteins, CD63, Alix, and Flotillin-1, confirming successful isolation of EVs.

To validate the possibility of molecular profiling of tumor EVs enriched by TASPMB, EVs were isolated from human breast cancer SKBR3 and colorectal cancer HT29 cell lines, as well as from blood plasma of a colorectal patient using the SEC method. All samples of EVs underwent characterization by different methods following the MISEV2023 criteria [32]. The mean and standard deviation of the hydrodynamic size distributions were determined by using nanoparticle tracking analysis (NTA) and were found to be 110 ± 58 nm, 109 ± 61 nm, and 122 ± 64 nm for HT29, SKBR3, and plasma EVs (Figure 2A). The concentrations of 1.62 ± 0.12 × 10^11^, 9.45 ± 0.41 × 10^10^, 6.60 ± 0.43 × 10^11^ were estimated for HT29, SKBR3, and plasma EVs, respectively. The mean protein concentration in HT29, SKBR3, and plasma EV samples was 105 ± 7 μg/mL, 96 ± 4 μg/mL, and 122 ± 12 μg/mL as determined by using the BCA method. (Figure 2B). Analysis of transmission electron microscopy (TEM) images of EVs depicted in Figure 2C showed that all EVs have a typical round shape morphology and phospholipid bilayer boundary. The size of EVs observed in TEM images is in good agreement with the results obtained by the NTA method. Further, EV samples were inspected by the Western blotting (WB) method. Fraction F9 to F13 of each cell line were analyzed in order to evidence the isolation of EVs. We also inspected fraction F14 to F20 to further confirm successful EV enrichment. The tetraspanins CD63, CD9, and CD81 were highly expressed fractions F9 to F13 collected both from HT29 and SKBR3 cell lines (Figure 2D and Figure S1). In contrast, CD63 and CD81 expression was low in fractions F14 to F20, while CD9 was absent in these fractions (Figure S1). The presence of two characteristic EV proteins, Alix and Flotillin-1, was detected in cell-derived EVs, while EpCAM expression was low (Figure S2). Lysed HT29 and SKBR3 cells were used as control samples for immunoblotting of Calnexin to verify sample purity (not found in EVs) (Figure S1). CD63, Alix, Flotillin-1, and EpCAM were highly expressed in plasma EVs (Figure 2D, Figures S1 and S2). Further, the presence of negative EV markers, such as ApoA1, ApoB, and IgG in plasma samples was also analyzed. The blots indicated that serum proteins were present in plasma-derived EVs; however, the amount of the proteins was lower compared to the crude plasma (Figure S3).

### 2.4. Capture of EVs and Detection of Their Membrane Proteins

**Figure 3:**
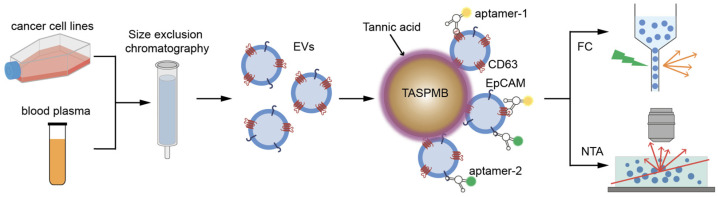
Schematic drawing of molecular profiling of EVs on tannic acid-coated superparamagnetic beads (TASPMB). EVs are first enriched from human breast cancer SKBR3 and colorectal cancer HT29 cell lines, and blood plasma of a colorectal cancer patient using a size-exclusion chromatography (SEC) column. EVs are then captured on TASPMB through incubation of TASPMB and EVs for 1 h at RT. The number of adsorbed EVs on TASPMB is determined by the nanoparticle tracking analysis (NTA) method, counting EVs in the supernatants. TASPMB-EV complex is incubated with either anti-CD63 or anti-EpCAM aptamers and analyzed by flow cytometry (FC). The orange and red arrows in the FC and NTA diagrams represent scattered light.

The protocol for capturing EVs by TASPMB, followed by analysis of their membrane proteins using flow cytometry (FC), is illustrated in Figure 3. The developed assay enables the simultaneous monitoring of the number of trapped EVs and identification of EV membrane proteins.

**Figure 4:**
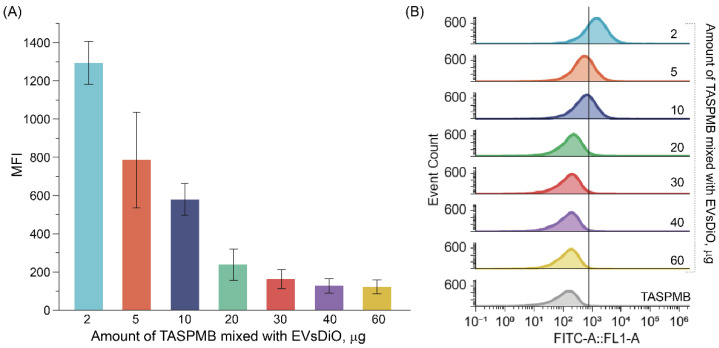
Optimization of the amount of tannic acid-coated superparamagnetic beads (TASPMB) for EVs capture. EVs isolated from a human colorectal cancer HT29 cell line containing a lipophilic dye (3,3′ dioctadecyloxacarbocyanine (DiO)) in the membrane were incubated for 1 h at RT with TASPMB (from 2 to 60 μg). (**A**) Median fluorescence intensity (MFI) of TASPMB with adsorbed HT29-DiO EVs as function of amount of TASPMB incubated with HT29-DiO EVs. (**B**) Histograms showing the distribution of DiO for different amounts of TASPMB incubated with HT29-DiO EVs. Error bars indicate the standard deviation (SD) (*n* = 3); *n* is the number of independent experiments.

To find the optimal amount of TASPMB and ensure reliable quantification of EVs, we first tested the capture efficiency of dye-labeled EVs [33]. The mean standard deviation and hydrodynamic size distribution of HT29-DiO EVs were measured in scattered and fluorescence modes using ZetaView PMX420-QUATT. The proportion of fluorescent HT29 EVs from the total number of EVs was about 5.5% (Figures S4 and S5). Fluorescence signal of HT29-DiO EVs with a maximum fluorescent intensity at 511 nm wavelength was also observed, confirming successful HT29 EVs labeling with lipophilic dye (Figure S6). TASPMB and HT29-DiO EVs were mixed with different amounts of TASPMB (from 2 to 60 μg). The number of unbound HT29-DiO EVs present in the supernatant was determined by the NTA method and these numbers were subtracted from the original concentration of HT29-DiO EVs added to TASPMB. Inspection of the samples using FC revealed that the fluorescence signal of HT29-DiO EVs on TASPMB decreases as the amount of beads changes (Figure 4). Median fluorescence intensity (MFI) was high at 2 μg of TASPMB and then gradually decreased as the amount of TASPMB reached 20 μg. Adding more TASPMB does not change fluorescence, and the signal is close to background. Inspection of supernatants of the samples revealed that capture efficiency is the same for all amounts of TASPMB added to EVs. The capture efficiency was found to be about 95% (Figure S7). From these results, one can conclude that even 2 mg of TASPMB is enough to trap a high number of EVs.

**Figure 5:**
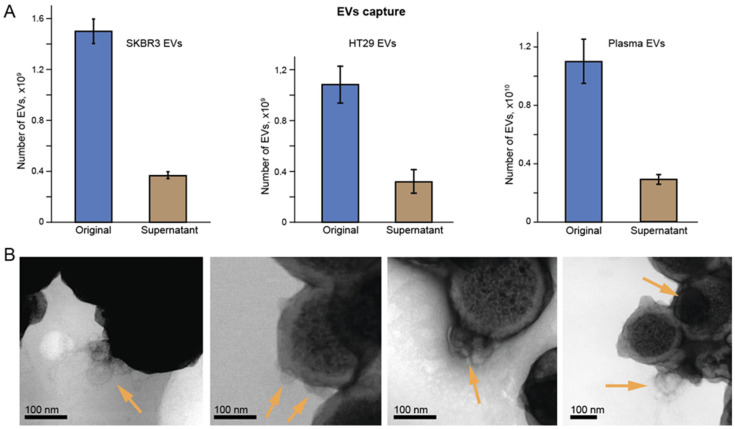
Capture of EVs on tannic acid-coated superparamagnetic beads (TASPMB). EVs derived from human breast cancer SKBR3 and colorectal cancer HT29 cell lines, and from blood plasma of a colorectal patient using the size-exclusion chromatography (SEC) method. (**A**) Histograms demonstrated the original concentration of EVs and the number of EVs in the supernatants. SKBR3, HT29, and plasma EVs were incubated with TASPMB using the ratio 1:10 for 1 h at room temperature (RT). (**B**) Set of transmission electron microscopy (TEM) images of TASPMB with adsorbed SKBR3 EVs. Arrows in TEM images indicate EVs on the surface of TASPMB. To increase the topographic contrast of EVs, the samples were treated with uranyl acetate. Error bars indicate the standard deviation (SD) (*n* = 3); *n* is the number of independent experiments.

Given the above results, we continued our study on labeling EV proteins with either anti-CD63 or anti-EpCAM aptamers, as depicted in the schematic of Figure 3. Figure 5A shows original concentrations of EVs before incubation with TASPMB and the number of EVs determined in the supernatants of each sample after collecting a TASPMB-EV complex. The average capture efficiency was determined to be 72% for all samples, which is in good agreement with our previous results [28]. Subsequently, TASPMB with adsorbed EVs were inspected with TEM, which confirmed successful capture of EVs by TASPMB. EVs on the surface of TASPMB maintain their round shape morphology with the characteristic boundary of the phospholipid bilayer (Figure 5B and Figure S8).

**Figure 6:**
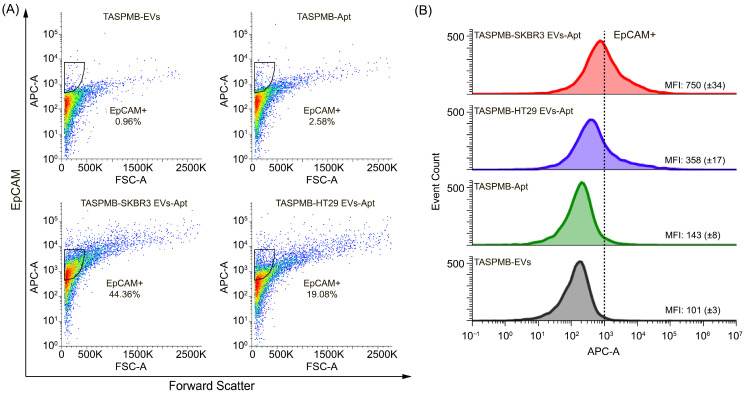
(**A**) Flow cytometry plots represent gating for EpCAM+ in EVs trapped by TASPMB. TASPMB-EV and TASPMB-Apt are used as controls. Gating is set based on TASPMB-EVs in order to eliminate false positive events due to aggregates (black area). The colors in the plots represent the density of EpCAM+ EVs, with blue representing lower EV density and red representing higher EV density. (**B**) Overlay of flow cytometry histograms showing EpCAM expression (APC fluorescence signal) for TASPMB-SKBR3 EVs, TASPMB-HT29 EVs, and controls. MFI (median fluorescence intensity) values for all samples are given in each histogram. Values in brackets indicate the standard deviation (SD) (*n* = 3); *n* is the number of independent experiments.

Thereafter, labeling of EV membrane proteins was performed by incubating the TASPMB-EV complex with either anti-CD63 or anti-EpCAM aptamers. TASPMB with EVs, and TASPMB incubated with either anti-CD63 or anti-EpCAM aptamers, were used as controls. Figure 6A shows EpCAM expression in SRBR3 and HT29 EVs adsorbed on TASPMB. The gates for TASPMB with SKBR3 and HT29 EVs have 44% and 19% EpCAM+ EVs. TASPMB with EVs showed low fluorescence intensity, while the gate for TASPMB incubated with anti-EpCAM aptamer is positioned just above max intensity of the population for TASPMB. This indicates very low non-specific binding and background fluorescence. Figure 6B displays an overlay of flow cytometry histograms illustrating the distribution of APC fluorescence intensity corresponding to EpCAM expression for the above samples compared to control samples. It is found that EpCAM median fluorescence intensity (MFI) was higher for SKBR3 EVs compared to HT29. This is somewhat opposite to WB analysis of these samples, where high EpCAM expression was detected for plasma EVs, but not for HT29 and SKBR3 EV samples (Figure S2).

**Figure 7:**
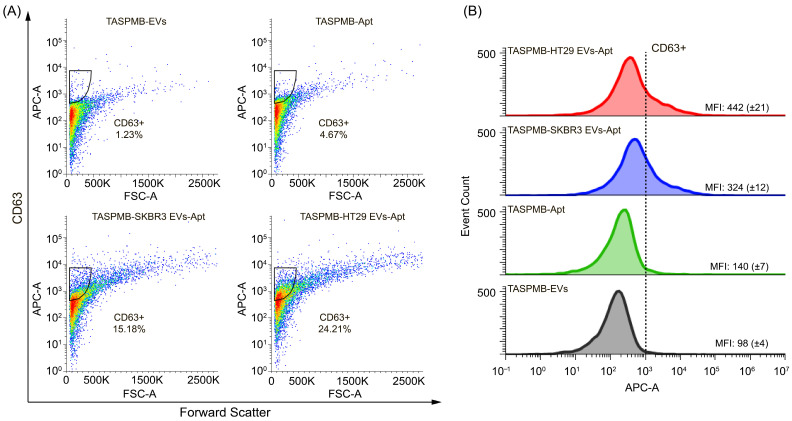
(**A**) Flow cytometry plots demonstrate gating for CD63+ in EVs absorbed on TASPMB. TASPMB-EV and TASPMB-Apt are used as controls. Gating is set based on TASPMB-EVs in order to eliminate false positive events due to aggregates (black area). The colors in the plots represent the density of CD63+ EVs, with blue representing lower EV density and red representing higher EV density. (**B**) Overlay of flow cytometry histograms of CD63 expression (APC fluorescence signal) for TASPMB-SKBR3 EVs, TASPMB-HT29 EVs, and controls. MFI (median fluorescence intensity) values for all samples are given in each histogram. Values in brackets indicate the standard deviation (SD) (*n* = 3); *n* is the number of independent experiments.

Subsequently, CD63 expression was identified in TASPMB-captured EV samples (Figure 7A). It is found that the gates for SKBR3 and HT29 EVs have 15% and 24% CD63+ EVs. Control sample TASPMB incubated with anti-CD63 aptamer showed low gate signal (~5%), which is just above the maximum signal for TASPMB-EVs. The distribution of APC fluorescence in FC histograms (corresponds to CD63 expression) is demonstrated in Figure 7B. One can see that HT29 EVs have higher MFI APC fluorescence in comparison with SKBR3 EVs. High intensity bands for CD63 protein in HT29 EVs was detected by WB (Figure 2D and Figure S1).

**Figure 8:**
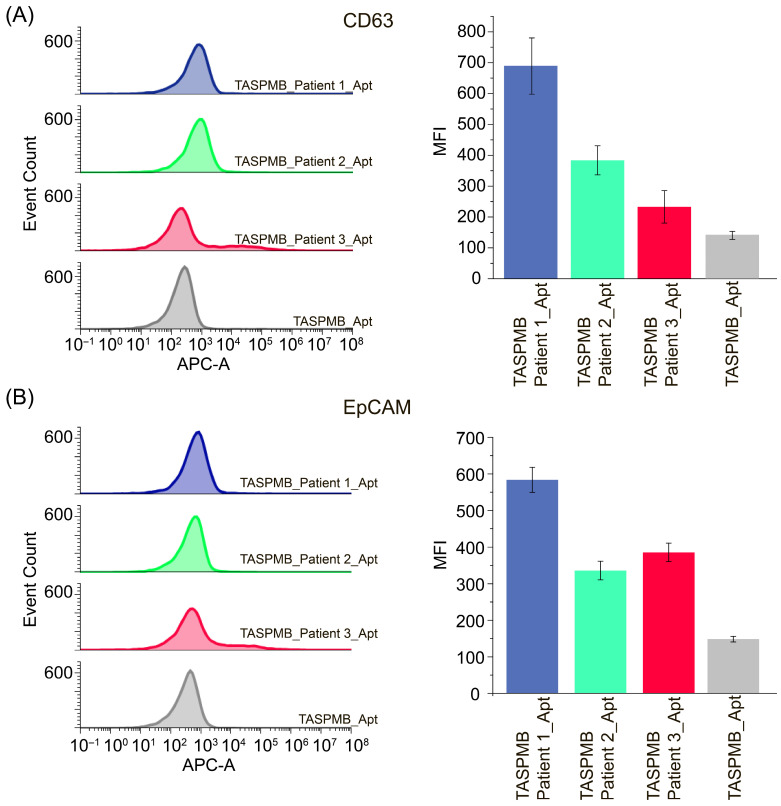
Histograms demonstrating CD63 (**A**) and EpCAM (**B**) expression for TASPMB + plasma EVs and control (**left**). The corresponding MFI (median fluorescence intensity) values for all samples are also shown (**right**). Error bars indicate the standard deviation (SD) (*n* = 3); *n* is the number of independent experiments.

Figure 8 demonstrates CD63 and EpCAM expression in EVs adsorbed on TASPMB. One can see that expression of both markers is deviated in cohort of patients. Higher MFI APC fluorescence of both markers was observed for patient 1, while the other two MFI values for these markers were lower. Although WB analysis revealed high CD63 and EpCAM proteins expression for plasma EVs, APC fluorescence signals of these proteins for TASPMB-captured plasma EVs were relatively low compared with HT29 and SKBR3 EVs. This is attributed to the presence of serum proteins such as albumin and apolipoproteins in plasma samples (detected by WB in plasma EVs, Figure S3) enriched using the SEC method combined with ultrafiltration [34]. These contaminating proteins, together with EVs, can competitively bind to tannic acid and hence reduce the number of EVs attached to TASPMB. Moreover, plasma proteins can form a “corona” layer on the EV membrane, suggesting that EV membrane proteins might be blocked by protein corona, which in turn would prevent aptamer binding and efficient detection by flow cytometry [35].

**Figure 9:**
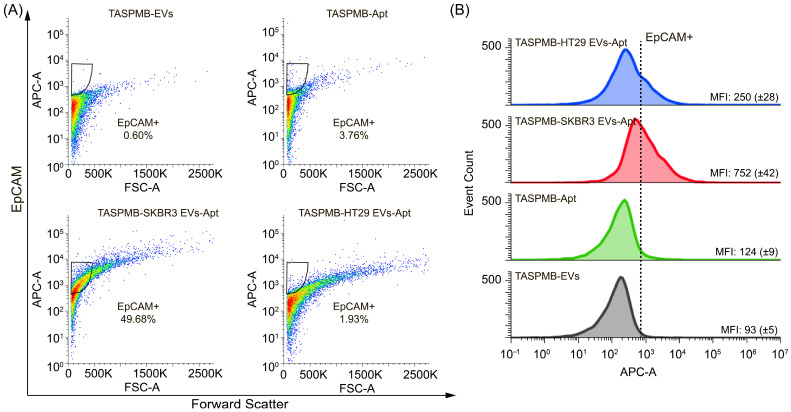
(**A**) Flow cytometry plots show gating for EpCAM+ EVs trapped by TASPMB from conditioned media of HT29 and SKBR3 cell cultures without pre-enrichment using SEC column. TASPMB-EVs and TASPMB-Apt are used as controls. The colors in the plots represent the density of EpCAM+ EVs, with blue representing lower EV density and red representing higher EV density. (**B**) Overlay of flow cytometry histograms showing EpCAM expression (APC fluorescence signal) for TASPMB-SKBR3 EVs, TASPMB-HT29 EVs, and controls. MFI corresponds to median fluorescence intensity and is given in each histogram. Values in brackets indicate the standard deviation (SD) (*n* = 3); *n* is the number of independent experiments.

**Figure 10:**
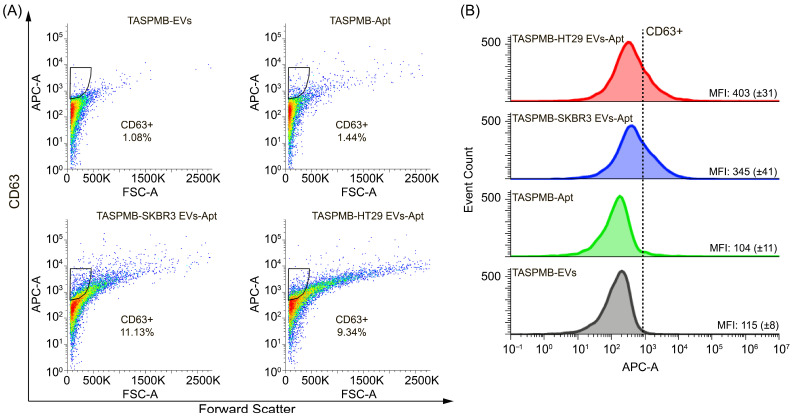
(**A**) Flow cytometry plots show gating for CD63+ EVs trapped by TASPMB from conditioned media of HT29 and SKBR3 cell cultures without pre-enrichment using SEC column. TASPMB-EVs and TASPMB-Apt are used as controls. The colors in the plots represent the density of CD63+ EVs, with blue representing lower EV density and red representing higher EV density. (**B**) Overlay of flow cytometry histograms showing CD63 expression (APC fluorescence signal) for TASPMB-SKBR3 EVs, TASPMB-HT29 EVs, and controls. MFI corresponds to median fluorescence intensity and is given in each histogram. Values in brackets indicate the standard deviation (SD) (*n* = 3); *n* is the number of independent experiments.

Furthermore, to verify whether our approach can be applied to the molecular profiling of EVs enriched by TASPMB from biological samples, we tested the labeling of membrane proteins on EVs isolated by TASPMB from the conditioned media of HT29 and SKBR3 cell cultures. Notably, at this stage, isolation of EVs from cell medium was conducted by only TASPMB without preliminary enrichment by the SEC method. Figure 8A represents expression of EpCAM for HT29 EVs and SKBR3 EVs versus control TASPMB and TASPMB-Apt samples. It is seen that the gate for SKBR3 EVs was 49%, while the gate for HT29 EVs was just above TASPMB-EVs. The control TASPMB-Apt sample has very low fluorescence background, indicating low non-specific binding. Increased MFI of EpCAM was found for SKBR3 EVs, which is about three times higher in comparison with EpCAM MFI of HT29 EVs (Figure 9B). CD63 expression in HT29 and SKBR3 EVs trapped from conditioned media is given in Figure 10A. Here we found the gate value of 9% of CD63 protein in HT29 EVs. The gate for SKBR3 EVs was just above (11%). HT29 EVs were shown higher CD63 MFI against for SKBR3 sample (Figure 10B).

We also analyzed the presence and expression levels of exosome-specific markers such as CD9, CD63, and CD81 by WB in lysates of EVs enriched on TASPMB (Figure S1). The CD9, CD63, and CD81 bands were faint, most probably due to the low number of EVs present in the samples. The absence of bands for Calnexin indicates absence in the samples of cellular components. Despite the low band intensity of exosome proteins determined by WB in lysate samples, FC allows detection of exosome-specific markers on EVs enriched on TASPMB, even at low numbers of EVs present on TASPMB.

## 3. Discussion

This study introduces a novel technique that employs tannic acid-coated superparamagnetic beads in combination with conventional flow cytometry for the efficient isolation and characterization of membrane proteins in extracellular vesicles. The approach was first verified on EVs, which were pre-isolated from human cancer cell lines and a colorectal cancer patient by SEC. We optimized EV:TASPMB ratio and the buffer composition to obtain a reliable fluorescence intensity for the identification of EV membrane proteins. These findings allowed us to successfully test for phenotyping of EVs isolated from cell culture medium by TASPMB without SEC enrichment. The proposed method capitalizes on the unique properties of tannic acid, which forms robust, non-specific bonds with phospholipids and proteins that make up the EV membrane, resulting in the formation of a highly stable complex of TASPMB and EV. A high association between EV and TASPMB appears to be advantageous when washing the TASPMB-EV complex, which likely results in additional purification of trapped EVs. This, in turn, liberates binding sites on the EV membrane and enables efficient molecular profiling of EVs using dye-labeled aptamers. In our previous study, we demonstrated that the size of EVs in both the supernatant and the eluate remained unchanged, suggesting that EVs adsorbed onto magnetic beads are purified from the proteins contained in the samples [28]. Therefore, we believe that contaminating protein can be washed off the surface of the beads before flow cytometry analysis.

Despite the lack of specific trapping of EVs through individual proteins, this approach achieves a remarkable capture efficiency (~72%), enabling the precise identification and quantification of membrane-associated proteins by flow cytometry. Compared with existing affinity-based capture systems, the tannic acid-based coating has several advantages: it is cost-effective, simpler to implement, and scalable using the layer-by-layer assembly method. Additionally, the surface modification can be conducted rapidly on-site without requiring specialized equipment, making TASPMB suitable for clinical applications.

However, while promising, this platform requires further optimization before it can be widely adopted. Current limitations include weak fluorescent signals when detecting membrane-bound proteins in real samples, necessitating the development of improved experimental protocols, such as optimized buffer compositions and enhanced labeling strategies. Buffer compositions play an important role in maintaining optimal pH and ionic strength for efficient binding during the experiment. Additionally, an optimal buffer composition helps preserve the integrity of bound ligands and prevents degradation of proteins or magnetic beads through aggregation. Potassium is one of the main cations in the cellular environment and can significantly affect the structure of nucleic acids, including RNA aptamers. Potassium can stabilize certain secondary structures, which can lead to a decrease in the affinity of the aptamer for the target molecules. In our studies, we used buffered solutions without potassium, which makes it possible to eliminate undesirable conformational changes in aptamers and ensure reliable binding of vesicles to membrane proteins. We also added Tween-20 due to two reasons. Tween-20 acts as a detergent, reducing the surface tension of solutions. This reduces the likelihood of non-specific protein adsorption on the surface of magnetic bits. The use of small concentrations of Tween-20 (usually about 0.01–0.1%) reduces the aggregation of macromolecules. This increases the reproducibility and reliability of the analysis. Enhanced labeling strategies require simpler and faster protocols, which will decrease the cost and duration of experiments. Modification of the surface of magnetic beads with tannic acid via simple and cost-effective LbL method for EVs capture is an attractive new strategy as the final step in sample preparation before labeling and analysis. Moreover, further optimization studies for clinical samples should be concentrated on the following points: (i) dilute versus concentrated plasma samples due to presence of high amounts of immunoglobulins and other vesicles. It is assumed that working with diluted samples can avoid absorbance of contaminating proteins on the beads and thus increase the efficiency of EVs’ interaction with the platform; (ii) variation in the amount of EVs released by each patient due to their particular pathophysiological conditions. Addressing these hurdles could improve the sensitivity and reproducibility of analyses, ultimately facilitating the reliable and accurate detection of biomarkers within liquid biopsies derived from EV populations.

## 4. Materials and Methods

### 4.1. Materials

Phosphate-buffered saline (PBS, pH 7.4, HIMEDIA, Thane, India). Ethanol (J.T. Baker, Phillipsburg, NJ, USA). Lipophilic carbocyanine dye 3,3′dioctadecyloxacarbocyanine (DiO, Lumiprobe, Westminster, MD, USA). 3-aminopropyltriethoxysilane (APTES, Sigma-Aldrich, St. Louis, MO, USA). Uranyl acetate dihydrate (Sisco Research Laboratories, Mumbai, Maharashtra, India). Bovine serum albumin (BSA, ≥96%, ~66 kDa, Sigma-Aldrich, USA). Tannic acid (TA, residue on ignition ≤0.5%, Sigma-Aldrich, St. Louis, MO, USA). SDS-PAGE gel, nitrocellulose membranes, and blocking buffer (Bio-Rad, Hercules, CA, USA). Primary monoclonal antibody specific to CD63 (Cell Signaling Technology, Boston, MA, USA, Rabbit mAb #10112), CD81 (Cell Signaling Technology, Rabbit mAb #56039), CD9 (Cell Signaling Technology, Rabbit mAb #13174), Alix (Cell Signaling Technology, Rabbit mAb #92880), Flotillin-1 (Cell Signaling Technology, Rabbit mAb #18634). Primary monoclonal antibody anti-EpCAM (PrimeBioMed, Moscow, Russia, VU-1D9, Mouse Ab, #10-310017-01). Primary antibodies specific to apolipoprotein A1 (RAH Laa, Imtek, Moscow, Russia), B (RAH Lbb, Imtek) and IgG (MGH Igg, Imtek). Primary antibody specific to Calnexin (Cloud-Clone Corp., Katy, TX, USA, Rabbit #PAA280Hu01). Secondary antibodies conjugated with HRP against rabbit immunoglobulin (Bio-Rad, Hercules, CA, USA, #1706515). Secondary antibodies conjugated with HRP against mouse immunoglobulin (Sorbent, Moscow, Russia, Mouse Ab #1069). Cyanine5 NHS ester (Cy5)-labeled anti-EpCAM aptamer (5′-ag-tga-cgc-agc-atg-cgg-cac-aca-ctt-cta-tct-ttg-cgg-aac-tcc-tgc-gg-(Cy5), Synthol, Moscow, Russia). Cyanine5 NHS ester (Cy5)-labeled anti-CD63 aptamer (5′-ta-acc-acc-cca-cct-cgc-tcc-cgt-gac-act-aat-gct-aat-tcc-aa-(Cy5), Synthol, Russia). BCA protein assay kit (FineTest, Wuhan, China). JetSpin™ Centrifugal Filter (15 mL, 100,000 MWCO and 5 mL, 50,000 MWCO, Jet Bio-Filtration, Guangzhou, China). Size-exclusion chromatography PURE-EVs (HBM-PEV-5) and mini PURE-EVs (HBM-mPEV-10) columns (HansaBioMed, Tallinn, Estonia). Protein LoBind tubes (Eppendorf, Hamburg, Germany). Deionized water purified on a Millipore Milli-Q system (Merck, Darmstadt, Germany) with resistivity 18 MΩ·cm was used in the experiments. All chemicals were used without further purification.

### 4.2. Preparation of Superparamagnetic Beads

#### 4.2.1. Synthesis of Hydrophobic Iron Oxide Nanoparticles

A mixture of 5 mmol of iron(II) chloride dihydrate and 10 mmol of iron(III) chloride hexahydrate in 50 mL of deionized water was slowly added to 200 mL of 1 M ammonia solution under vigorous stirring at 80 °C in an argon atmosphere. Then, 1 mL of oleic acid was added, and the stirring continued for 15 min. The black precipitate was separated by a neodymium magnet and washed several times with distilled water and acetone. The colloid was then redispersed in chloroform, forming a total concentration of iron oxide of 10 mg/mL.

#### 4.2.2. Synthesis of Silica-Coated Superparamagnetic Beads

The emulsion droplet solvent evaporation method was employed for the fabrication of iron oxide clusters, as described in the protocol reported in [36], with minor modifications. An amount of 1 mL of hydrophobic iron oxide nanoparticles was added to 1 mL of 60 mM myristyltrimethylammonium bromide solution in deionized water and sonicated for 5 min. Then the mixture was transferred into a three-necked flask and heated at 60 °C for one hour with argon purification to evaporate the organic solvent. The prepared clear solution of iron oxide clusters is then redispersed in 3 mL of 3 mM poly(vinylpyrrolidone) solution in ethylene glycol and stirred for 5 min. The resulting polymer-capped iron oxide clusters were precipitated with a magnet and washed three times with deionized water. The silica shells were synthesized by the standard Stöber method. An amount of 10 mg of iron oxide clusters was transferred to 50 mL of 90% ethanol containing 0.02 mmol of ammonia, sonicated for 5 min, followed by adding 0.1 mL of tetraethoxysilane. The reaction mixture was stirred vigorously at room temperature for 1 h. The black precipitate of silica-coated iron oxide clusters was then collected by a magnet and washed thrice with ethanol and water.

#### 4.2.3. Functionalization of Silica-Coated Iron Oxide Clusters with Amino Groups

An amount of 10 mg of silica-coated iron oxide clusters was resuspended in 30 mL of ethanol, and then 0.5 mL of APTES was added under an argon atmosphere with vigorous stirring. The resulting mixture was then kept at 60 °C for 6 h. After the reaction had stopped, the beads were washed three times with ethanol to remove.

### 4.3. Deposition of Tannic Acid on Amino-Functionalized Silica-Coated Superparamagnetic Beads

An amount of 1.6 mL of BSA water solution (2 mg/mL) was added to 0.2 mL aqueous suspension of amino-functionalized silica-coated iron oxide clusters (1 mg), followed by agitation on a shaker for 15 min. The clusters were then washed three times with water to remove unbound protein molecules. Then, 1.6 mL of TA water solution (2 mg/mL) was added to the cluster suspension and agitated for another 15 min, followed by washing three times with water. The deposition of BSA and TA was repeated five times, which resulted in the following composition on the cluster surface: Clusters/(BSA/TA)5. The clusters were kept in water at 4 °C for further use.

### 4.4. Characterization of Superparamagnetic Beads (SPMB)

To characterize the SPMB during surface functionalization with amino groups and deposition of polyelectrolytes, several methods were used. Scanning electron microscopy (SEM) images of the nanoparticles were obtained using a Quattro microscope (Thermo Fisher Scientific, Waltham, MA, USA). Transmission electron microscopy (TEM) images were obtained using a Titan microscope (Thermo Fisher Scientific, USA) equipped with an EDX attachment (USA). Energy dispersive X-ray analysis (EDS) was performed using a transmission electron microscope equipped with attachments for elemental analysis using energy dispersive X-ray spectroscopy (Super-X EDX detector) and electron energy loss spectroscopy (Quantum 965) methods. Zeta potential was determined by electrophoretic light scattering (ELS) on a Zetasizer Nano ZS instrument (Malvern Panalytical, Westborough, MA USA).

### 4.5. Isolation of EVs from Cell Culture and Blood Plasma

In the present study, EVs were derived from human breast cancer SKBR3 cell lines and human colorectal cancer HT29 cell lines. Cell lines were grown in 175 cm^2^ culture flasks in DMEM medium with 10% fetal bovine serum (FBS), 5% L-Glutamine, 1% sodium pyruvate, 1% penicillin/streptomycin, and 0.1% gentamicin. After reaching a confluence of 90–100%, the culture medium was replaced with a medium without FBS for 48 h before the collection of EVs. After 48 h, the culture medium was collected and centrifuged at 400× *g* for 10 min to remove dead cell debris. The samples of culture medium were frozen and stored at −20 °C in 50 mL Eppendorf tubes. Before EV isolation by ultrafiltration (UF) and size-exclusion chromatography (SEC) methods, cell culture medium (~90 mL volume for each cell line) was sequentially centrifuged 300× *g* for 10 min, 1200× *g* for 20 min to remove aggregates, concentrated, and preliminary purified by using JetSpin™ Centrifugal 100 kDa filter. To isolate EVs, 500 μL of concentrated cell culture medium was loaded in the precleaned mini PURE-EVs SEC column, and five 100 μL fractions were collected according to the protocol provided by the manufacturer.

The blood sample was a donation from N.N. Blokhin National Medical Research Center of Oncology within protocol #9 dated 18.09.2023. Cells were removed from plasma by centrifugation at 1000× *g* and 4 °C for 10 min. The supernatant was then centrifuged again at 2000× *g* and 4 °C to remove platelets, aliquoted into 2 mL tubes, and stored at −20 °C before EV isolation. To isolate EVs from plasma, 1 mL of thawed sample was diluted in 1 mL of DBPS and centrifuged for 30 min at 4500× *g* at 4 °C, and 2 mL of supernatant was loaded into the prepared PURE-EVs SEC column. Three 500 μL fractions enriched in EVs were collected according to the protocol provided by the manufacturer.

Samples of EVs derived from cell cultures and plasma were stored at −20 °C in protein LoBind tubes until further analysis.

HT29 EVs were isolated four times from four HT29 cell line samples, SKBR3 EVs were obtained three times from three SKBR3 cell line samples, and plasma EVs were isolated twice from 2 mL crude plasma of patients.

### 4.6. Conjugation of EVs with Lipophilic Dye

Fluorescently labeled EVs were obtained using the following procedure: 200 μL of isolated EVs from HT29 cell culture medium (average number of vesicles 2 × 10^10^ particles/mL) in DPBS was loaded in a protein LoBind tube and two μL of DiO dye (2 uM in a DMSO:PBS mixture (1:1)) was injected to the tube followed by incubation of the tube in a shaker at RT for 5 min. To remove dye excess, the sample was then loaded in the prepared SEC column and five 100 μL fractions collected according to the protocol provided by the manufacturer. DiO-labeled EVs were stored at −4 °C in LoBind tubes until further analysis.

### 4.7. Characterization of EVs

To evaluate the efficiency and purity of isolated EVs, EV samples were characterized by using transmission electron microscopy (TEM), nanoparticle tracking analysis (NTA), analysis of total protein with bicinchoninic acid (BCA), and Western blotting (WB).

Before TEM imaging, samples of isolated EVs were transferred to deionized water to minimize the amount of salt crystals formed upon drying and concentrated to a final volume of 200–250 μL by using JetSpin™ Centrifugal 50 kDa filter. A carbon grid was treated on a glow discharge system to make the film hydrophilic. Then, the grid was incubated with a droplet (~20 μL) of the EV sample for 10 s. The excess of the EV sample was removed by blotting the grid perpendicular to the filter paper. The grid was then incubated on a drop of uranyl acetate water solution (1.5 mg/mL) for 30 s, followed by blotting the stain away using filter paper and drying the grid for 10 min before imaging. The grid was fixed on the TEM sample holder, and EVs were analyzed at magnification ranging from 60,000 to 340,000 times by using Titan Themis Z TEM (Thermo Fisher Scientific, Waltham, MA, USA).

Protein amount in EV samples was determined by using the micro BCA protein assay according to the manufacturer’s protocol. EV samples (100 μL) were incubated with an equal volume of the working reagent for 2 h at 37 °C. Absorbance was measured at 562 nm by using an Infinite Nano+ plate reader (Tecan Group Ltd., Männedorf, Switzerland). The analysis was performed for three replicates.

The size and concentration of EVs were estimated by using a ZetaView^®^ PMX420-QUATT instrument (Particle Metrix, Inning am Ammersee, Germany). The laser wavelength used in scatter and fluorescent modes was 488 nm, and the filter wavelength used in fluorescent mode was 520 nm. Samples with isolated EVs were diluted 1:80–1:2000 in DPBS before video recording. The recording was repeated five times for each sample. The videos were analyzed using ZetaView software (version 8.05.14 SP7) to determine the size distribution of EVs, their mean, and EV concentration.

For Western blotting, EV samples were separated on the SDS-PAGE gel and electrotransferred to nitrocellulose membranes. Membranes were washed with PBST (0.1% Tween-20 in PBS) and blocked for 30 min at RT in 5% nonfat milk (for Alix or Flotillin-1) or 5% BSA (for CD63, CD9, CD81, Calnexin or EpCAM) in PBST. The presence or absence of CD63, CD9, CD81, Calnexin, Alix, Flotillin-1, and EpCAM was determined on separate membranes, which were incubated overnight at 4 °C with primary antibodies diluted 1:500 in blocking buffer while shaking gently. In the case of ApoA, ApoB1 and IgG plasma used for isolation of EVs was first diluted in PBS to achieve total protein concentration (Protein A280) the same as plasma EV samples. A 4% gel was used for ApoB, while 15% gel was used for ApoA and IgG. Primary antibodies used for ApoA, ApoB1, and IgG analysis were diluted 1:1000. After five washes, 5 min each, with PBST solution, membranes were incubated in a blocking buffer containing peroxidase-labeled secondary antibodies diluted 1:2500 (1:5000 in case of ApoA, ApoB1, and IgG) for 1 h at room temperature. The membranes were washed five times with PBST and developed using SuperSignal™ West Femto Maximum Sensitivity Substrate (Thermo Fisher Scientific, Waltham, MA, USA) according to the manufacturer’s instructions. Immunoreactive bands were visualized using the MicroChemi 4.2 Imaging System (DNR Bio Imaging System, Jerusalem, Israel).

An amount of 100 μL of EVs-DiO sample was added to a 96 plate, and fluorescence spectrum was acquired at 430 nm excitation wavelength in emission wavelength interval from 475 to 600 nm with emission wavelength step size of 2 nm by using an Infinite Nano+ plate reader (Tecan Group Ltd., Switzerland). EV fractions from 8 to 14 were placed separately (2 μL) to a nitrocellulose membrane and dried before measurements. Measurements were performed at excitation and emission wavelengths of 460 nm and 520 nm by using the IVIS Spectrum CT imaging system (PerkinElmer, Shelton, CT, USA).

### 4.8. Capture of EVs by Tannic Acid-Coated Superparamagnetic Beads (TASPMB) and Detection of EV Membrane Proteins

First, SPMB were incubated with DiO-stained EVs, varying the ratio of SPMB and EV-DiO (1:25; 1:10; 1:5) for 1 h on a thermoshaker at 25 °C using 800 rpm. After the incubation, MB with bound EV-DiO were collected using a magnetic stand, and the supernatant containing unbound EV-DiO was transferred to a new protein, LoBind, for determining their concentration using the NTA method.

To estimate the amount of bound sEVs on the surface of tannic acid-coated beads, we calculated the capture efficiency (CE) using the following expression:

CE = ((N_int_ − N_sup_)/N_int_) × 100%

where N_int_ is the initial concentration of sEVs mixed with tannic acid-coated beads, and N_sup_ corresponds to the number of sEVs determined in the supernatant after the collection of SPMB. The N_int_ and N_sup_ concentrations of sEVs were determined using the nanoparticle tracking analysis (NTA) method.

Subsequently, TASPMB with attached EV-DiO was washed thrice with DPBS and fluorescence signal was immediately acquired from the TASPMB-EV-DiO complex by using CytoFLEX flow fluorescence cytometry (Beckman Coulter, Brea, CA, USA). The fluorescent signal was recorded in the FITC channel (ex: 488 nm; em: 520 nm).

To perform profiling of EVs pre-isolated by SEC from cell culture medium and plasma samples, 270 μL of EVs and 30 μL of TASPMB were mixed in a new protein LoBind tube, and the mixture was incubated for 1 h on a thermoshaker at 25 °C using 800 rpm. After the incubation, MB with trapped EVs were collected on a magnetic rack, and the supernatant was taken and transferred to a new protein LoBind tube to determine unbound EVs by using the NTA method. SPMB-EV complex was washed three times with DPBS and then resuspended in 400 μL of a buffer (150 mM NaCl, 10 mM Na_2_HPO_4_, 10 mM NaH_2_PO_4_) containing 0.01% Tween-20, and 100 μL of either anti-EpCAM aptamer (1 pM) or anti-CD63 aptamer (1 pM) was added to TASPMB-EV complex. The mixture was incubated for 30 min on a thermoshaker at 800 rpm and room temperature (RT). TASPMB-EV-Aptamer complex was collected on a magnetic stand, and the complex was washed several times with a buffer (150 mM NaCl, 10 mM Na_2_HPO_4_, 10 mM NaH_2_PO_4_) containing 0.01% Tween-20 to remove unbound aptamer molecules. The MB-EV-Aptamer complex was resuspended in 500 μL of a buffer (150 mM NaCl, 10 mM Na_2_HPO_4_, 10 mM NaH_2_PO_4_) and analyzed by using CytoFLEX cytometer.

To implement molecular profiling of EVs directly from cell culture medium, 20 μL of MB was mixed with 180 μL of conditioned media of SKBR3 or HT29 cell cultures and incubated on a thermoshaker at 25 °C using 800 rpm. After the incubation, TASPMB-EVs complex was washed thrice with DPBS and transferred to a 400 μL buffer (150 mM NaCl, 10 mM Na_2_HPO_4_, 10 mM NaH_2_PO_4_) containing 0.01% Tween-20. Then, 100 μL of either anti-EpCAM aptamer (1 pM) or anti-CD63 aptamer (1 pM) was added to the TASPMB-EV complex, and the mixture was incubated on a thermoshaker at 800 rpm and room temperature (RT). After 30 min of incubation, the TASPMB-EV-Aptamer complex was collected on a magnetic stand and thoroughly washed in a buffer (150 mM NaCl, 10 mM Na_2_HPO_4_, 10 mM NaH_2_PO_4_) containing 0.01% Tween-20 to purify it from unbound aptamer molecules. The SPMB-EV-Aptamer complex was transferred to a 500 μL buffer (150 mM NaCl, 10 mM Na_2_HPO_4_, 10 mM NaH_2_PO_4_) and measured on the CytoFLEX cytometer. The fluorescent signal from Cy5 labeled anti-EpCAM and anti-CD63 aptamers was captured in the APC channel (ex: 638 nm, em: 660 nm).

Flow cytometry data were analyzed with the free online software Floreada.io (https://floreada.io/).

## 5. Conclusions

In summary, we have successfully fabricated superparamagnetic beads and subsequently functionalized the bead surface with TA using the LbL deposition method. The beads permit the capture of EVs and the identification of EV membrane proteins by using conventional flow cytometry. We successfully profiled EVs derived from human cancer cell lines and a plasma cancer patient using aptamers against the most typical exosomal CD63 and cancer EpCAM proteins. The approach is also validated for EVs enriched by the beads from conditioned media of two cancer cell cultures without preliminary purification using the SEC method. We believe that this approach can be applied for molecular profiling of different types of EV proteins using various antigens, such as aptamers, antibodies, peptides, and others.

## Data Availability

The datasets used in the current study might be shared upon reasonable request to corresponding author.

## Supplementary Materials

The following supporting information can be downloaded at: https://www.mdpi.com/article/10.3390/ijms262311324/s1.

## Abbreviations

The following abbreviations are used in this manuscript:

EVs	Extracellular vesicles
FC	Flow cytometry
TASPMB	Tannic acid coated superparamagnetic beads.
TA	Tannic acid
SPMB	Superparamagnetic beads
SPIONs	Superparamagnetic iron oxide nanoparticles
SEC	Size-exclusion chromatography
NTA	Nanoparticles tracking analysis
LbL	Layer-by-layer
BCA	Bicinchoninic acid
TEM	Transmission electron microscopy
SEM	Scanning electron microscopy
WB	Western blotting
EpCAM	Epithelial cell adhesion molecule
