# Supplementary material for: Profiling of CD63 and EpCAM Membrane Proteins of Extracellular Vesicles on Tannic Acid-Coated Magnetic Beads Using Conventional Flow Cytometry
Source: Int J Mol Sci. 2025 Nov 23;26(23):11324. doi: 10.3390/ijms262311324 (PMC12692446; doi:10.3390/ijms262311324)
Supplement: Supplementary file 1 [file ijms-26-11324-s001.zip › ijms-3937168-supplementary.pdf]

Supplementary Information

Article

Profiling of CD63 and EpCAM Membrane Proteins of Extracellular Vesicles on Tannic Acid-Coated Magnetic Beads Using Conventional Flow Cytometry

Ekaterina Moiseeva [1], Igor Sergeev [1], Vasiliy Chernyshev [1,2], Olga Zaborova [3], Daria Kohzevnikova [1], Alexander Yakovlev [4,5], Olesya Kuznetsova [6], Alexey Tryakin [6], Aleksei Komlev [7], Dmitry Gorin [1] and Alexey Yashchenok [1,*]

[1] Skolkovo Institute of Science and Technology, Skolkovo Innovation Center, 121205 Moscow, Russia; ekaterina.moiseeva@skoltech.ru (E.M.); igor.sergeev@skoltech.ru (I.S.); v_chernyshev@oparina4.ru (V.C.); d.kozhevnikova@skoltech.ru (D.K.); d.gorin@skoltech.ru (D.G.)

[2] National Medical Research Center for Obstetrics, Gynecology and Perinatology Named After Academician V.I. Kulakov, 117997 Moscow, Russia

[3] Faculty of Chemistry, Moscow State University, 119991 Moscow, Russia; olya_z_88@mail.ru

[4] Moscow Research and Clinical Center for Neuropsychiatry, 115419 Moscow, Russia; al_yakovlev@ihna.ru

[5] Department of Functional Biochemistry of Nervous System, Institute of Higher Nervous Activity and Neurophysiology, Russian Academy of Sciences, 117865 Moscow, Russia

[6] N.N. Blokhin National Medical Research Center of Oncology, 115522 Moscow, Russia; lessya.kuznetsova@gmail.com (O.K.); atryakin@gmail.com (A.T.)

[7] Faculty of Physics, Moscow State University, 119991 Moscow, Russia; alkomlev98@yandex.ru

* Correspondence: a.yashchenok@skoltech.ru

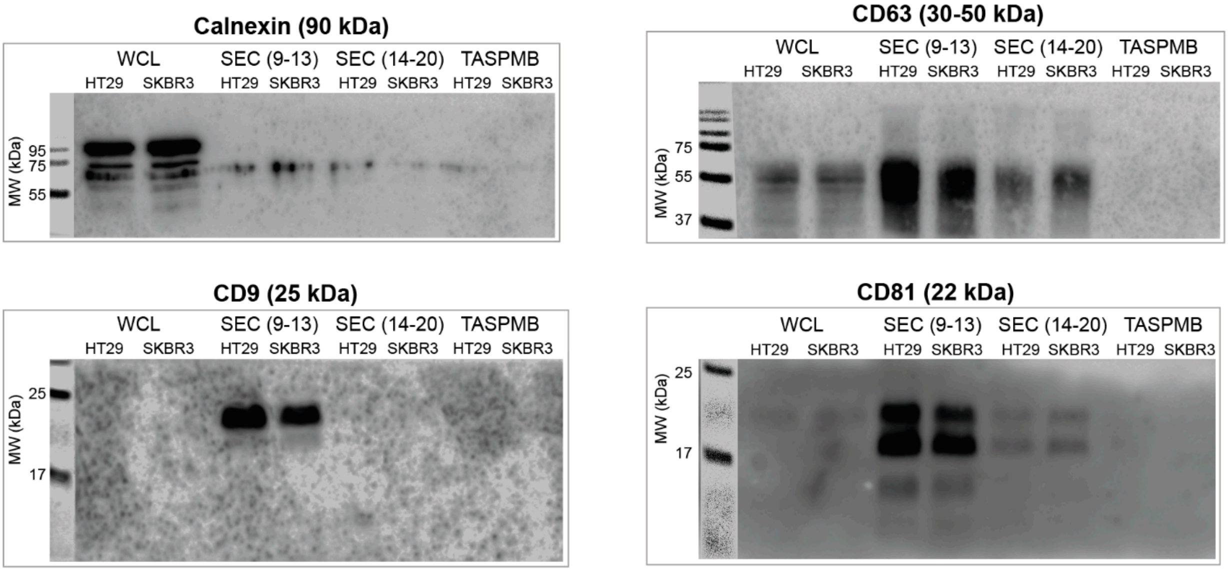

Figure S1. Immunoblotting of whole cell lysate (WCL) of HT29 and SKBR3 cell lines, EV samples derived from HT29 and SKBR3 cell lines (9-13 fractions collected in a SEC column), 14-20 fractions HT29 and SKBR3 cell media collected by a SEC column, and lysates from EVs enriched on TASPMB before flow cytometry analysis.

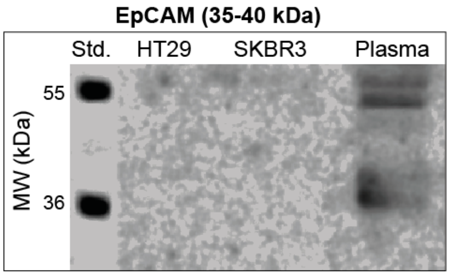

Figure S2. Immunoblotting of SKBR3, HT29, and plasma EVs demonstrates relative expression of EpCAM protein in each sample.

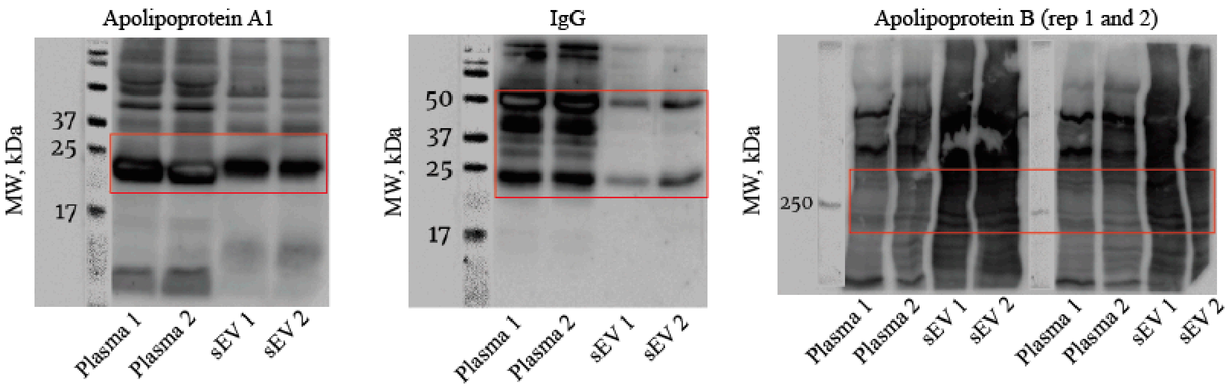

Figure S3. Immunoblotting of the crude plasma (Plasma 1 and Plasma 2) and EVs derived from plasma (sEV 1 and sEV 2) shows the presence of Apolipoprotein A1, Apolipoprotein B, and IgG in the samples.

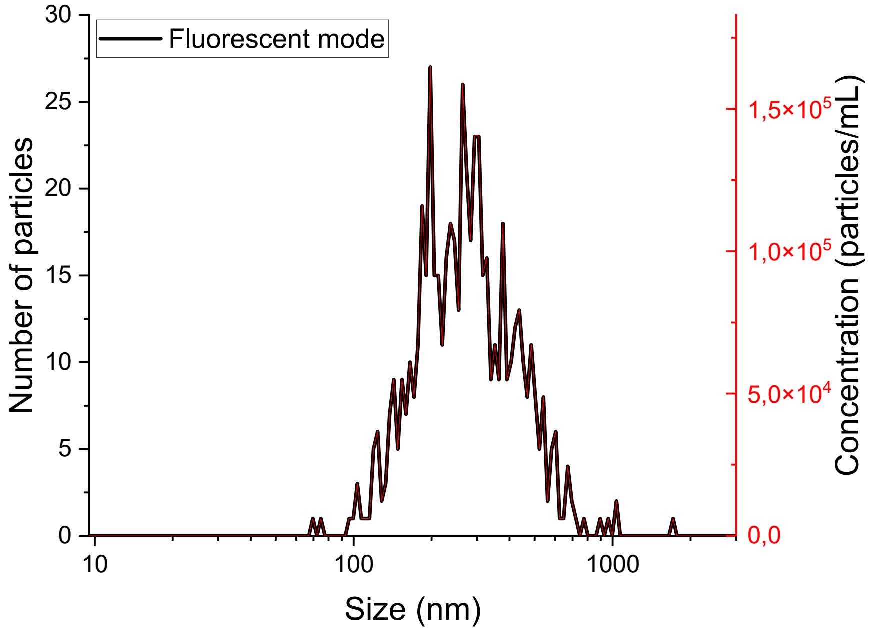

Figure S4. Hydrodynamic size distribution and concentration of HT29-DiO EVs (dilution 1:80 in PBS) measured by using nanoparticle tracking analysis (NTA) method in fluorescence mode. Laser wavelength 488 nm, filter wavelength 500 nm.

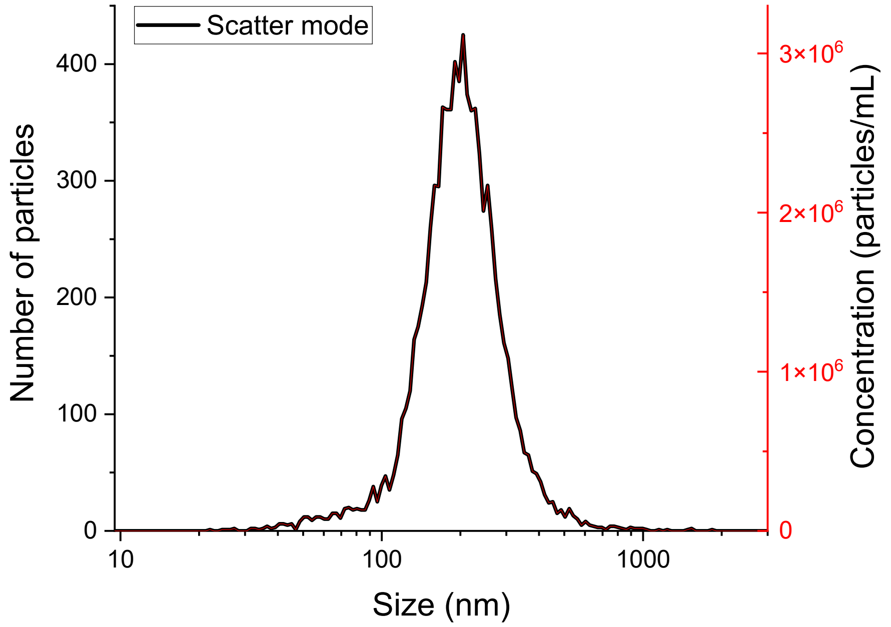

Figure S5. Hydrodynamic size distribution and concentration of HT29-DiO EVs (dilution 1:80 in PBS) measured by using nanoparticle tracking analysis (NTA) method in scatter mode. Laser wavelength 488 nm.

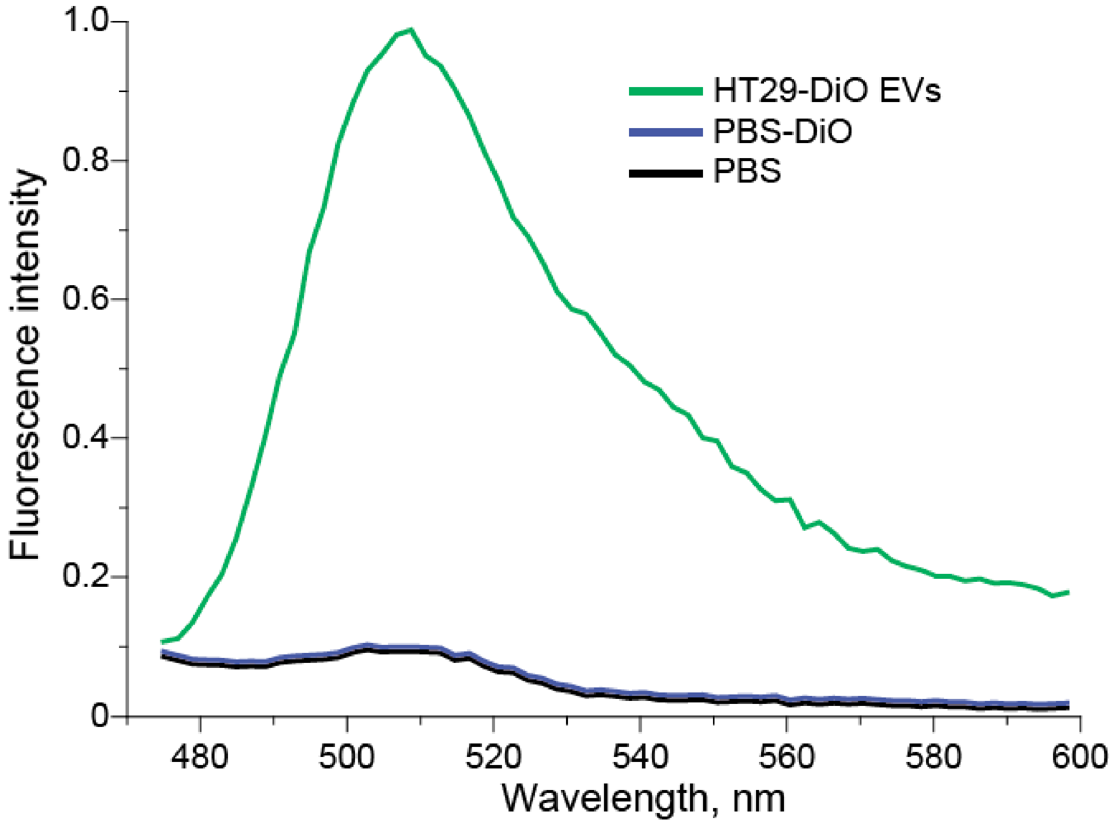

Figure S6. Fluorescence signal of 1.7 × 10^{10} HT29-DiO EVs in phosphate-buffered saline (PBS) (green curve). PBS was used as negative control (black curve). DiO dye of the same concentration incubated with PBS (PBS-DiO) at similar conditions as HT29-DiO EVs was used as positive control (blue curve). HT29-DiO EVs and PBS-DiO samples after incubation were purified on SEC column by collecting fractions corresponding to EVs' yield.

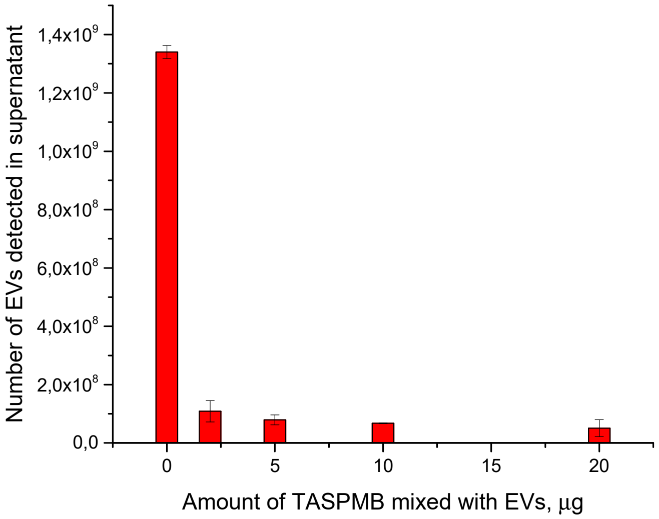

Figure S7. Number of HT29-DiO EVs determined in the supernatants after the incubation of EVs with different amount of TASPMB. To determine the concentration of EVs, the NTA method was used.

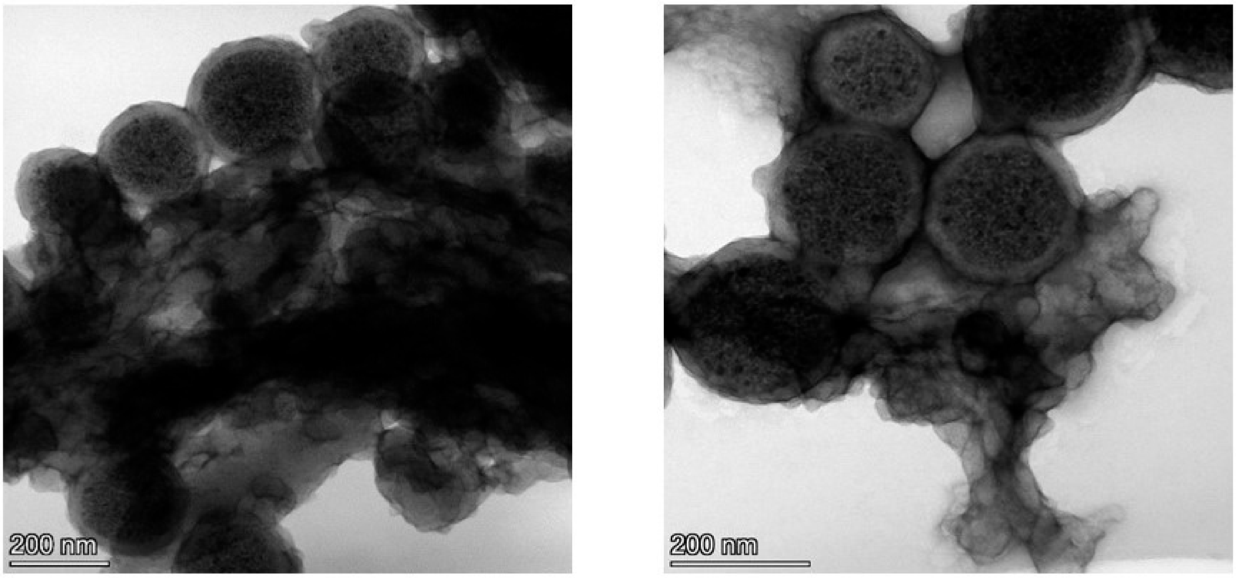

Figure S8. HRTEM images of TASPMB with adsorbed SKBR3 EVs.
